# Construction of a ferroptosis-based prediction model for the prognosis of MYCN-amplified neuroblastoma and screening and verification of target sites

**DOI:** 10.1186/s41065-025-00413-8

**Published:** 2025-03-19

**Authors:** Linjun Tan, Guoqian He, Chengqi Shen, Sijia He, Yan Chen, Xia Guo

**Affiliations:** 1 Department of Pediatrics, Affiliated Hospital of Zunyi Medical University, No.149 Dalian Road, Huichuan District, Zunyi, 563003 Guizhou China; 2 Department of Pediatrics, Guizhou Children’s Hospital, No.149 Dalian Road, Huichuan District, Zunyi, 563003 Guizhou China; 3 Department of Pediatrics, West China Second University Hospital, Sichuan University, Section 3, South Renmin Road, Chengdu, 610041 China; 4 Collaborative Innovation Center for Tissue Injury Repairand Regenerative Medicine of Zunyi Medical University, No.149 Dalian Road, Huichuan District, Zunyi, 563003 Guizhou China

**Keywords:** Neuroblastoma, Ferroptosis, MYCN, Prognosis, Prognostic score model

## Abstract

**Background:**

Neuroblastoma (NB) is a prevalent extracranial solid tumor in pediatric patients. Of these, the MYCN-amplified type has a poor treatment response and prognosis. To enhance therapeutic efficacy and prognostic outcomes, numerous research teams have undertaken extensive investigations through various pathways and directions. Among these, ferroptosis has recently emerged as a significant area of research focus.Ferroptosis, a type of iron-dependent cell death, is primarily caused by lipid peroxides. This study intends to develop a prognosis model based on MYCN-amplified NB and ferroptosis-related genes (FGs).

**Methods:**

Data for this study were sourced from the TARGET and FerrDb databases. Lasso regression algorithms and univariate COX analysis were leveraged to determine feature genes; multivariate COX analysis was employed to develop a prediction model and risk scores; and receiver operating characteristic (ROC) curves and Kaplan-Meier analysis were utilized to assess the predictive ability of the model. Furthermore, discrepancies in immune cell infiltration (ICI) between the high-risk (HR) and low-risk (LR) populations were assessed via CIBERSORT analysis. Finally, experiments were conducted on MYCN-amplified and MYCN non-amplified cells so as to validate the differential expression of the gene.

**Results:**

A prediction model was constructed and risk scores were calculated based on 4 genes (LIFR, TP53, NRAS, and OSBPL9). The HR group, which was stratified by the median score, had a lower overall survival rate than the LR group.The differences in expression of each gene between MYCN-amplified and MYCN non-amplified cells were further confirmed through cell experiments and qPCR.

**Conclusion:**

The prediction model in this study can be employed to forecast the prognosis of MYCN-amplified NB. These genes may represent promising new ferroptosis-related intervention targets (FITs) in treating MYCN-amplified NB, with the potential to improve patient outcomes.

**Supplementary information:**

The online version contains supplementary material available at 10.1186/s41065-025-00413-8.

## Introduction

NB, a tumor of the peripheral nervous system, originates from the primitive neural crest and typically manifests in the paraspinal ganglia or adrenal medulla [1, 2]. MYCN amplification has been demonstrated to facilitate the malignant progression of NB and can serve as an independent poor prognosis factor, in addition to clinical factors [3, 4]. Given that MYCN amplification frequently occurs in HR cases with poor treatment responses, high mortality, and poor prognosis, the scientific community is dedicated to developing new treatment strategies for this type of NB.

Ferroptosis refers to a non-apoptotic process of programmed cell death, which was first identified via chemical screening by Stockwell Laboratory in 2012. The process is defined by the buildup of intracellular lipids and reactive oxygen species (ROS) in the presence of iron [5]. Several studies revealed that ferroptosis exhibited a critical function in inhibiting tumor growth (TGI) and killing tumor cells. Relevant studies on breast cancer [6], lung cancer [7], pancreatic cancer [8], hepatocellular carcinoma [9], and ovarian cancer [10] revealed that inducing ferroptosis may be a novel cancer therapeutic approach. NB, the most prevalent solid tumor in pediatric patients, has also been the subject of numerous ferroptosis-related studies. It has been shown that high MYCN expression can stimulate the expression of TFRC which encodes the transferrin protein receptor 1. This increases intracellular iron loading and LIP levels, resulting in ferroptosis and enhanced lipid peroxidation [11]. MYCN synergistically directs the expression of a variety of receptors, activates the systemic Xc-receptor/GSH pathway, and also becomes iron-dependent, predisposing NB cells to lipid peroxidation [12]. The homocysteine requirement of MYCN-amplified NB in childhood is met by transsulfuration and uptake. Once the uptake is restricted, ferroptosis can be triggered via the GSH pathway [13]. Nevertheless, studies examining FITs in MYCN-amplified NB are scarce. These studies demonstrate that MYCN amplification exhibits sensitivity to ferroptosis, suggesting that targeting and inducing ferroptosis may represent a promising therapeutic strategy for MYCN-amplified neuroblastoma. However, research on ferroptosis-related intervention targets in MYCN-amplified neuroblastoma remains limited at present.

In this study, the R language tool was leveraged to conduct bioinformatics analysis on the TARGET database, identify DEGs, and perform functional enrichment analysis (FEA) to gain insight into associated functional mechanisms. Additionally, a prognosis model related to MYCN and ferroptosis genes was developed. Through cohort analysis verification and nomogram construction, it was demonstrated that the prediction ability of the model is specific and sensitive. Furthermore, the differential expression of genes was verified in MYCN-amplified and MYCN non-amplified cells, demonstrating that the results aligned with the bioinformatics analysis. According to these findings, it is concluded that the ferroptosis-related prognosis model based on LIFR, TP53, NRAS, and OSBPL9 may be a robust tool for forecasting the prognosis of MYCN-amplified NB patients.

## Materials and methods

### Data collection

RNA-seq profiles, clinical pathology information, and survival data for 151 NBL samples were obtained from the TARGET database. Additionally, FRG data were sourced from FerrDb (www.zhounan.org/ferrdb/current/).

### Difference analysis

**Fig. 1:**
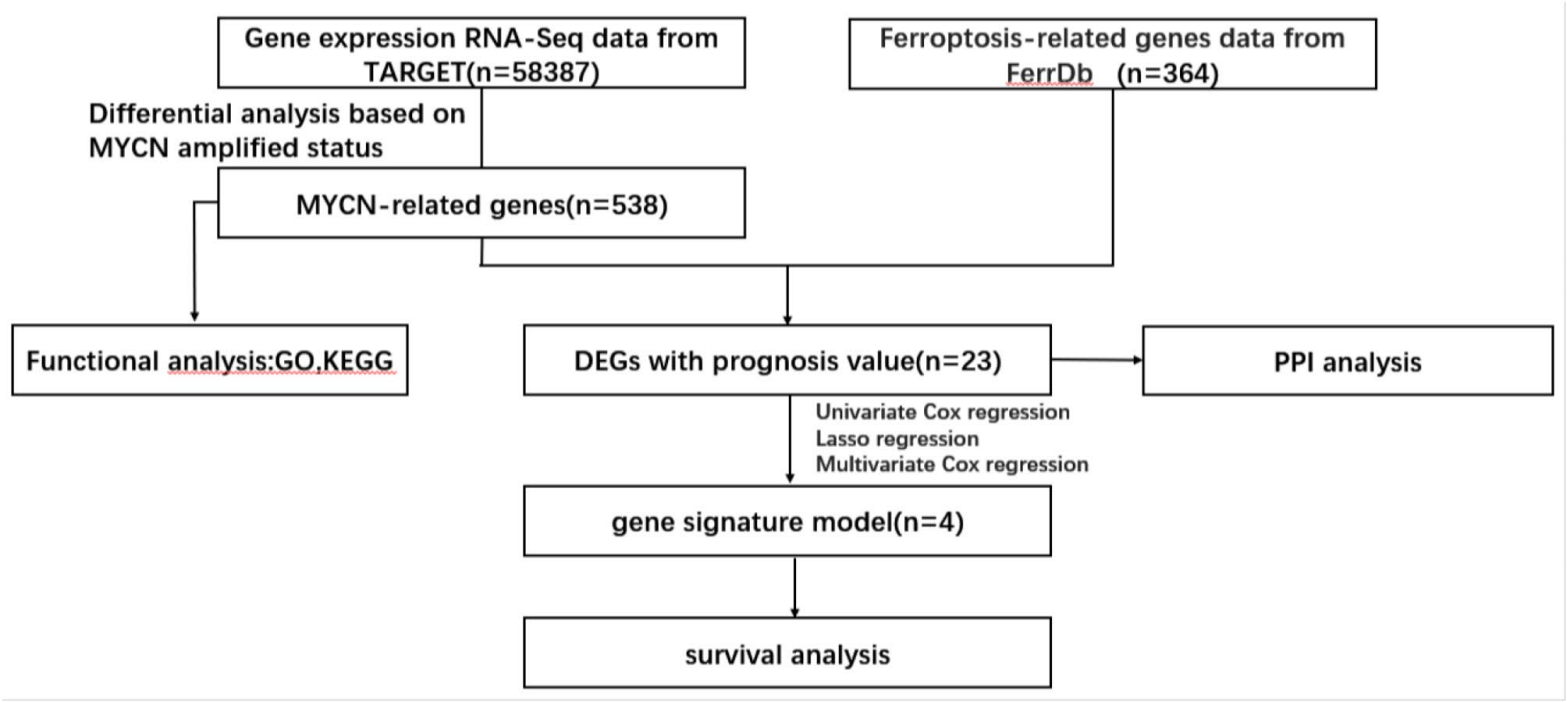
Bioinformatics analysis flowchart

Database samples were grouped according to MYCN amplification. They were initially screened using the “limma” R package in R language software (|log2FC| > 1.5, corrected p value < 0.05), and then the samples were crossed with FGs to identify DEGs related to MYCN amplification and ferroptosis. The study flowchart is illustrated in Fig. 1. The “VennDiagram”, “ggplot2”, and “pheatmap” R packages were employed to create Venn diagrams, volcano plots, and heat maps, respectively. The online STRING database was applied to generate an interactive network of DEGs.

### Construction of prognosis model and nomogram

Univariate COX analysis was executed to elucidate ferroptosis-related DEGs (FDEGs) related to the prognosis of MYCN-amplified NB. The “forestplot” R package in R was used to create a forest map, and Lasso regression analysis was leveraged to refine gene characteristics. The model’s penalty parameter (λ) was set in accordance with the minimum criterion (i.e., the value of λ corresponded to the lowest partial likelihood deviation). This was determined through ten-fold cross-validation, and then multivariate Cox regression model analysis was executed to identify the covariates of patient survival with independent prognostic values. On the basis of the normalized expression level and associated regression coefficients of each gene, a risk score was computed for each patient, and a prognosis model was constructed. The formula was established as: Risk score = ∑ (Coefi * Expi). The samples in the database were randomly divided into the training set and the testing set.Kaplan-Meier survival analysis was utilized for the comparison of the difference in overall survival (OS) between HR and LR groups in the training set and the testing set, respectively. The predictive ability was tested for the prognosis risk score model via ROC curves. The “ggrisk” R package in R was applied to visualize risk factors. Additionally, an independent dataset (GSE85047) from the GEO database was utilized for external validation. Patients were stratified into high-risk and low-risk groups based on the calculated risk scores, and survival curves along with survival status plots were subsequently generated. A range of comprehensive clinical factors such as gender, age, risk score, and stage were used as prognostic indicators. The “rms” R package was employed to develop a nomogram for forecasting the survival probability at 1, 3, and 5 years.

### FEA

The “clusterProfiler” R package in R was utilized to analyze the functional enrichment of significant DEGs, such as biological processes (BP), cellular components (CC), molecular functions (MF) of GO, and eight biological metabolic pathways analyzed by KEGG. P value was adjusted via the BH method.

### ICI analysis

The Estimation of STromal and Immune cells in MAlignant Tumour tissues using Expression data (ESTIMATE) tool is capable of calculating the matrix, immune, and ESTIMATE scores for each tumor sample, as well as the tumor purity. Cibersort [14] was employed to evaluate infiltrating immune cells (ICs) and the composition of different TMEs in HR and LR groups. Cibersort is a tool that uses a white blood cell (WBC) gene signature matrix, designated LM22, to ascertain the prevalence of 22 immune-related cells in a mixed-cell population. The “reshape2”, “ggplot2”, “ggthemes”, “RColorBrewer”, and “ggpubr” R packages were employed to plot.

### Cell culture and reagents

The MYCN-amplified human NB cell line SK-N-BE [2] and the MYCN non-amplified human NB cell line SH-SY5Y (Wuhan Pricella Biotechnology Co., Ltd., China) were cultured using SH-SY5Y dedicated complete medium and SK-N-BE [2] dedicated complete medium (Wuhan Pricella Biotechnology Co., Ltd., China) in a thermostatic cell incubator (PHCbi, Japan) at 37 °C with a CO2 concentration of 5%.

### Real-time fluorescence quantitative PCR

TRIzol (Thermo, USA) was employed to extract total RNA. Nanodrop2000 spectrophotometer (Thermo, USA) was utilized to measure RNA concentration. Transcriptor First Strand cDNA Synthesis Kit (Roche, USA) was leveraged for reverse transcription of RNA into cDNA, and then Taq Pro Universal SYBR Qpcr Master Mix Kit (Vazyme International LLC, China) was utilized to mix RNA with the cDNA. The qRCR process was conducted on a fluorescence quantitative PCR instrument (BIO-RAD, USA). The results were statistically analyzed via the 2-ΔΔCt algorithm, with GAPDH as the reference gene. The specific primer sequences for each gene and corresponding product lengths are presented in Table 1.

**Table 1:** PCR amplification primers and sequences

Primer Name	Location	Primer Sequence
GAPDH	Upstream	5’-GGAGCGAGATCCCTCCAAAAT-3’
	Downstream	5’-GGCTGTTGTCATACTTCTCATGG-3’
OSBOL9	Upstream	5’-GCGTCCATCTTCCCTACCAG-3’
	Downstream	5’-ACGTGGCTTGGAGAAGTGAG-3’
LIFR	Upstream	5’-TGGAACGACAGGGGTTCAGT-3’
	Downstream	5’-GAGTTGTGTTGTGGGTCACTAA-3’
TP53	Upstream	5’-GTGGTAATCTACTGGGACGGA-3’
	Downstream	5’-CTTTCTTGCGGAGATTCTCTTC-3’
NRAS	Upstream	5’-AAGTACTGTAGATGTGGCTCGC-3’
	Downstream	5’-AAGATGATCCGACAAGTGAGAG-3’

### Statistical analysis

A bioinformatics analysis was conducted via R 4.3.1, and the experiment was repeated three times. The statistical analysis was executed via GraphPad Prism 9. For comparing two sets of data, Student’s t-test was employed if the data conformed to a normal distribution; otherwise, Mann-Whitney U-test was adopted. In the figures presented in the result section, “*” indicates statistical significance (*p < 0.05, **p < 0.005, ***p < 0.0005, ****p < 0.0001), and ns represents that it is not statistically significant.

## Results

### Identification of MYCN-related DEGs and FDEGs

**Fig. 2:**
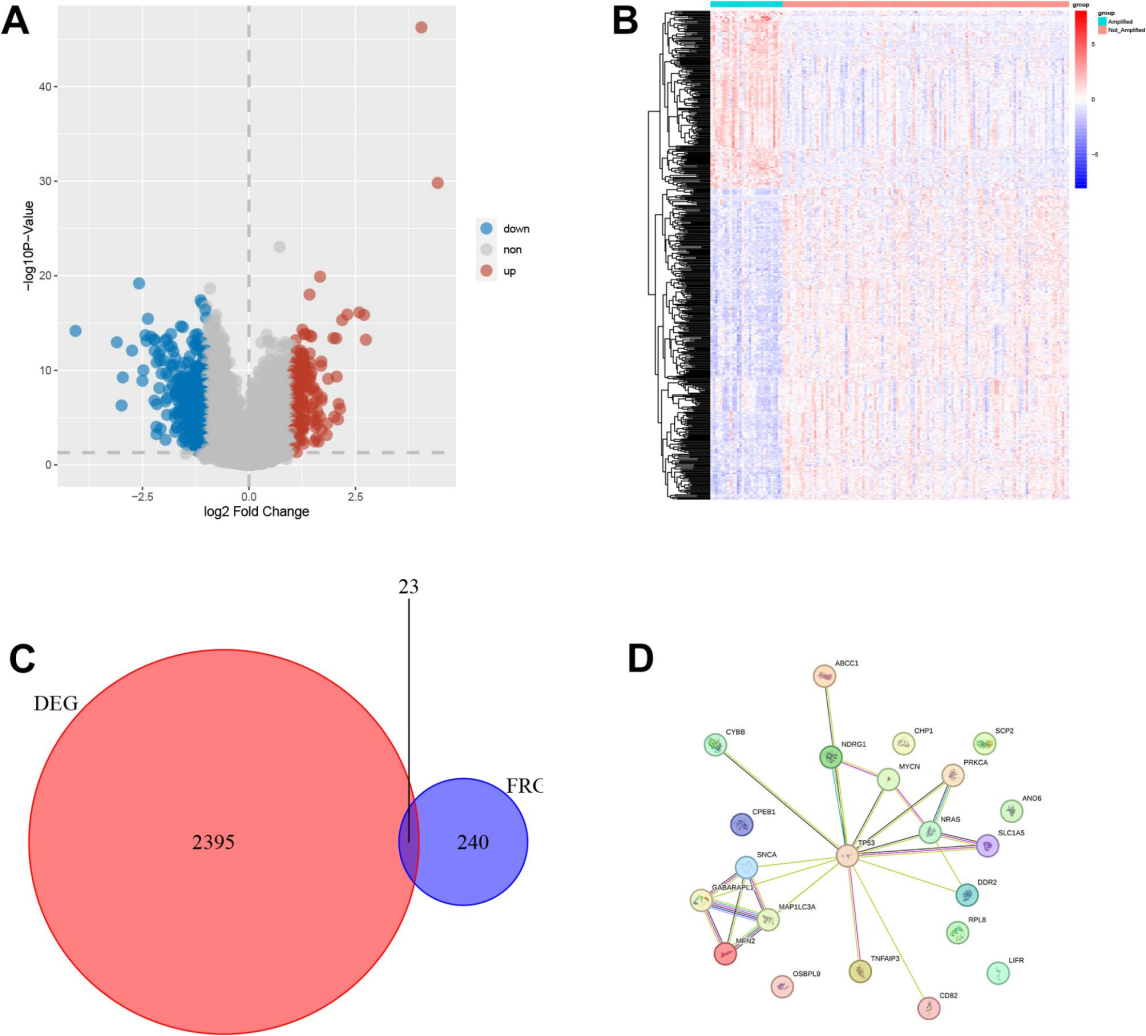
Prognostic DEGs correlated with MYCN and ferroptosis in TARGET Database (**A**) Volcano plot for DEGs correlated with MYCN amplification. The red dots represent genes that are upregulated in the NBL sample organization, the blue dots represent genes that are downregulated, and the gray dots represent genes with no significant differences (**B**) Heat map for DEGs correlated with MYCN amplification, used for visualizing the expression levels of genes (**C**) Venn diagram for FDEGs (**D**) Interaction between candidate genes in the PPI network. The TP53 gene, in addition to its association with MYCN, has functional connections with multiple genes, indicating the complex molecular mechanisms by which TP53 influences ferroptosis

RNA-seq maps and clinical information for 151 NBL samples in this study were sourced from the TARGET database. Following conversion, expression data on 58,387 genes were obtained, and data on 364 FRGs were downloaded from the FerrDb database. A differential expression analysis via the “limma” R package identified 538 DEGs associated with MYCN amplification (Fig. 2A and B).

After adjusting the appropriate fold change, 2395 differentially expressed genes related to MYCN amplification were obtained, and then intersected with 240 ferroptosis-related genes.The subsequent intersection with FGs yielded a total of 23 DEGs (Fig. 2C). The PPI network demonstrated the interconnection of these genes (Fig. 2D).

### Construction and validation of prognosis model

**Fig. 3:**
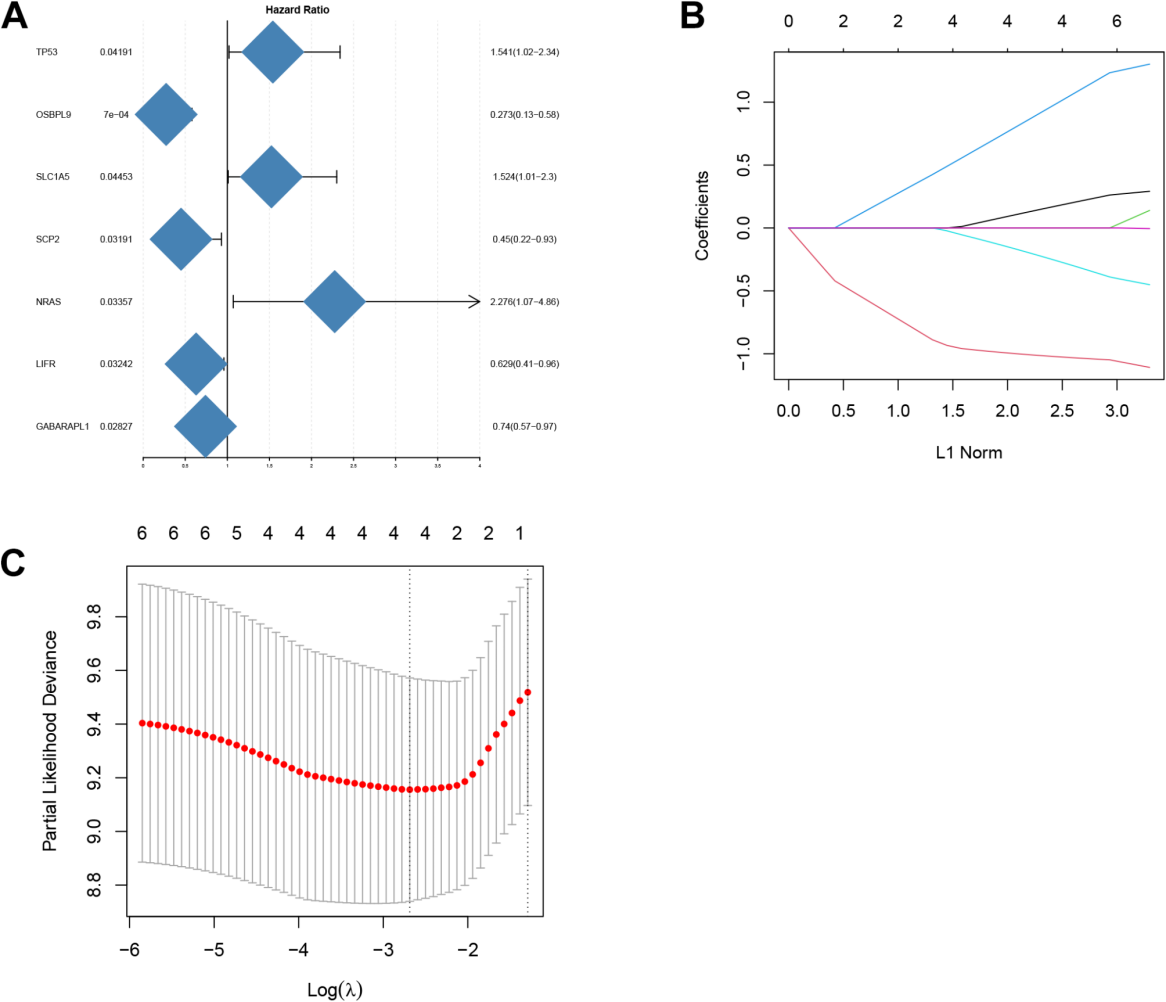
Key Genes in the training set (**A**) Forest plot for FDEGs correlated with prognosis in the training set by univariate COX analysis, HR(Hazard Ratio) > 1 is protective gene and HR < 1 is risk gene (**B**) Lasso regression analysis coefficient pathways of 7 FGs (**C**) Cross-validation curve of Lasso regression analysis, The dashed line represents lambda.min

**Fig. 4:**
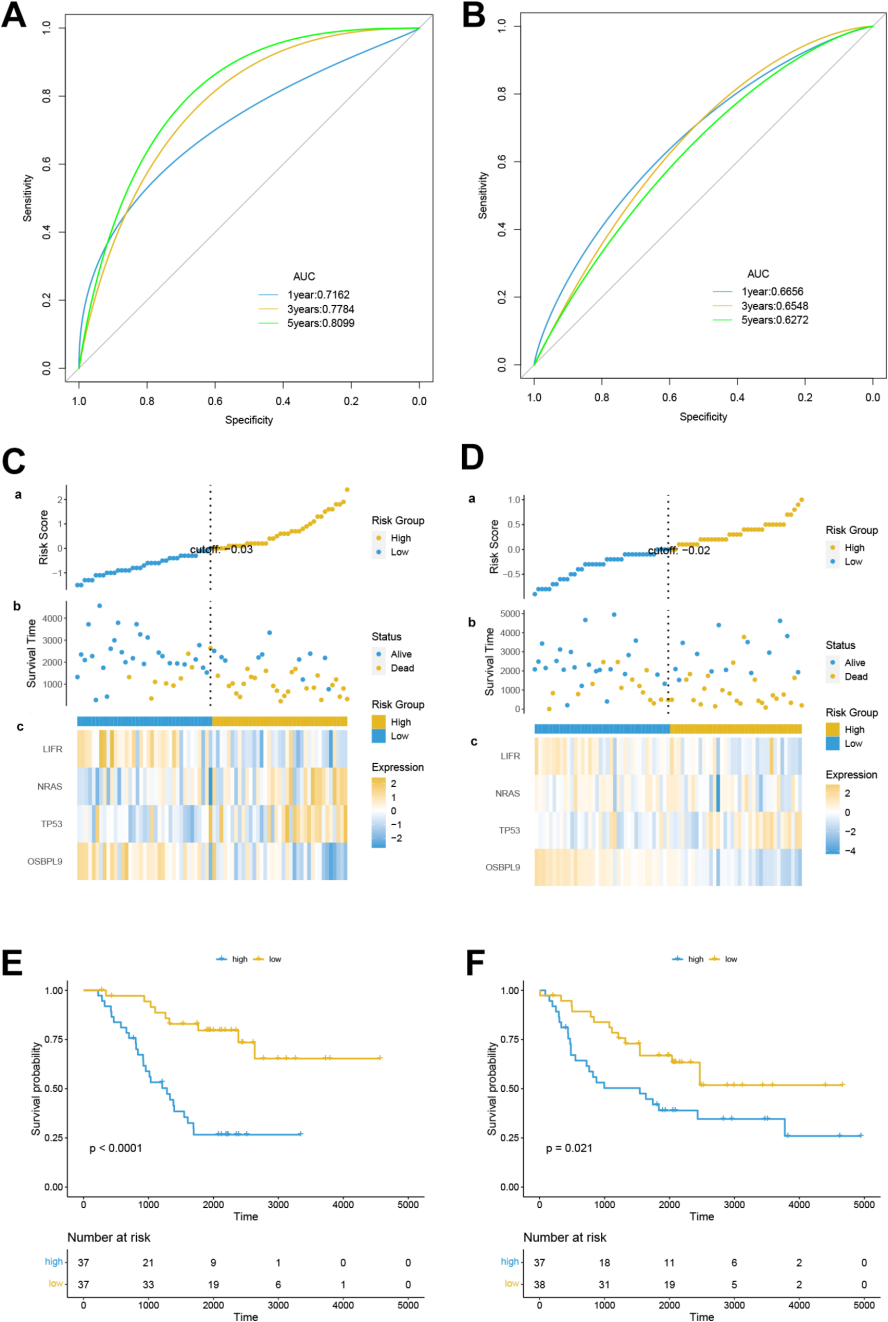
Validation of Model Prediction Effect in the Training Set and the Testing Set (**A**), (**B**) Validation of prognostic value of FRG features in the two sets (**C**), (**D**) Heat map for risk score distribution, survival overviews, and key genes in the two sets (**E**), (**F**) Kaplan-Meier overall survival curves for the HR and LR groups in the two sets. The survival outcomes of the low-risk group (blue) are significantly better than those of the high-risk group (yellow)

**Fig. 5:**
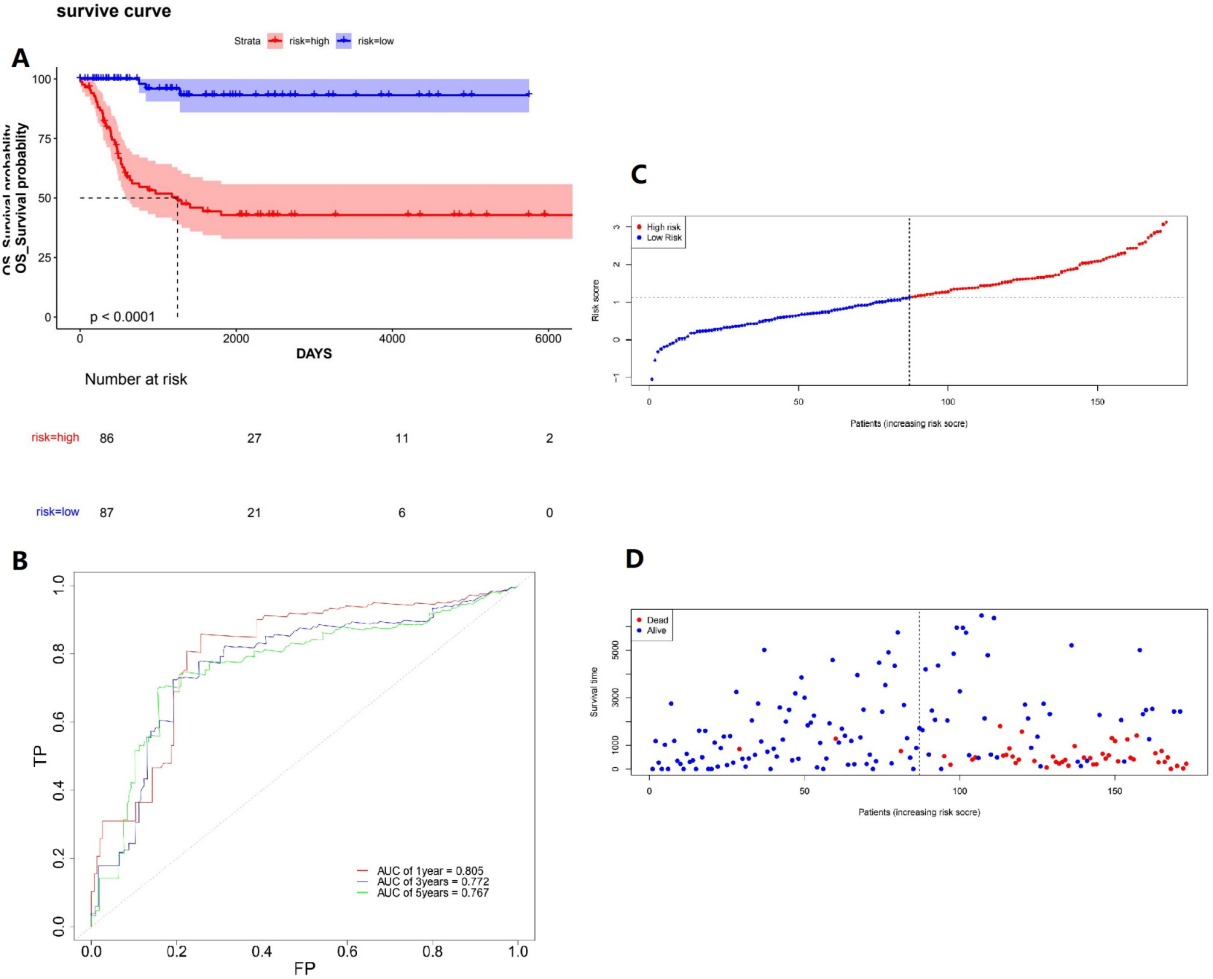
The GEO dataset is used to externally verify the prediction effect of the model (**A**) The overall survival curve of Kaplan-Meier showed that the survival rate of the low-risk group was significantly higher than that of the high-risk group (*p* < 0.0001) (**B**) ROC curves verify the prognostic value of FRG features in external datasets (**C**) distribution of risk scores per patient based on survival models (**D**) Based on the survival status of the two groups of patients based on the model score, more patients died as the risk score increased

In this study, 7 FDEGs related to prognosis were determined via univariate Cox regression analysis (Fig. 3A), and 4 key genes were further determined utilizing Lasso regression (Fig. 3B and C). Multivariate Cox regression analysis was then carried out, and a prognosis model was constructed based on the 4 genes. The formula: (-1.054 × OSBPL9 expression level) + (0.288 × TP53 expression level) + (-0.432 × LIFR expression level) + (1.311 × NRAS expression level) was employed to compute risk scores. All subjects were randomly assigned to the training set and the testing set. The predictive ability was assessed for risk scores via time-dependent ROC curves. The areas under the curve (AUCs) at 1, 3, and 5 years were 0.716, 0.778, and 0.809, respectively, in the training set (Fig. 4A), and 0.666, 0.655, and 0.627, respectively, in the testing set (Fig. 4B). These results demonstrated that the prognosis model had favorable predictive efficiency. Samples were arranged to HR and LR groups in the training set and the testing set, respectively, using the median as the critical value. The results revealed that the HR group had a higher mortality rate, the expression of LIFR and OSBPL9 was downregulated, and the expression of NRAS and TP53 was upregulated (Fig. 4C and D). Kaplan-Meier survival curve also unraveled that the HR group exhibited an OS rate lower than that in the LR group (Fig. 4E and F). To further validate the reliability of the predictive model, we conducted external validation by screening an independent dataset from the GEO database. Based on the median risk score, the NB samples within the dataset were stratified into high-risk and low-risk groups. Kaplan-Meier analysis demonstrated that the survival rate of the low-risk group was significantly higher than that of the high-risk group (*p* < 0.0001, Fig. 5A). The ROC curve revealed AUC values of 0.805, 0.772, and 0.767 for 1-year, 3-year, and 5-year survival, respectively (Fig. 5B). The distribution of risk scores and the visualization of overall survival (OS) status further indicated that the survival probability of patients in the high-risk group was significantly lower than that of patients in the low-risk group(Fig. 5C and D). Validation results based on the external NB sample dataset demonstrated that the predictive capability of our risk model is stronger than that observed in samples solely based on MYCN amplification.

### Independent prognosis prediction value of risk scores and construction of nomogram

Multivariate COX regression analysis demonstrated that risk scores identified in this study can serve as a prognosis predictor of NB independent of age, gender, stage, and MYCN amplification status (*P* < 0.05) (Fig S1A). Additionally, several clinical predictors were integrated to develop a nomogram for the prediction of 1-, 3-, and 5-year OS (Fig S1B).

### Functional enrichment analysis of MYCN-related DEGs

For more understanding of pathways and biological functions of DEGs (Figs S2A and S2B), this study executed a GO and KEGG functional enrichment analysis on MYCN-related DEGs. The GO results unraveled that DEGs were markedly enriched in nerve synaptic tissue and cell connections, and the KEGG results unveiled that DEGs were notably enriched in pathways related to COVID-19 and cell adhesion molecules.

### Assessment of immune microenvironment

The CIBERSORT method was adopted for further analyzing differences in immunoinfiltrating cells between the HR and LR groups, determining the types and composition ratios of 22 ICs in the tumor, and identifying any discrepancies in IC components between the HR and LR groups. According to the results, there were differences in the infiltration of only two IC types between the two groups: resting memory T cells CD4 and memory B cells. Notably, resting memory T cells CD4 showed a higher proportion in the LR group (Fig S3).

### Cell experiment verification

To further verify the differential expression of NRAS, OSBPL9, TP53, and LIFR between MYCN-amplified and MYCN non-amplified groups, this study selected MYCN-amplified SK-N-BE [2] cells and MYCN non-amplified SH-SY5Y cells for QPCR experimental verification. The experiment indicated that the expression of NRAS, OSBPL9, and LIFR was consistent with the results of the bioinformatics analysis, while the expression of the TP53 gene was opposite (Table S1, Figs S4).

## Discussion

NB, the most typical extracranial tumor in children, is a significant contributor to cancer-related fatalities in this population. The MYCN gene was identified as early as the 1980s and has been shown to significantly enhance the invasiveness of NB. Furthermore, the amplification state of this gene significantly reduces the survival rate of NB patients [15, 16]. Consequently, targeting MYCN represents the optimal treatment strategy and the most effective means of improving prognosis. Many molecular targeted therapies have been developed in recent years, such as induction of apoptosis, autophagy, and other cell death methods [17, 18]. Ferroptosis, a novel form of cell death, presents a promising avenue for enhancing the treatment and prognosis of various cancers. Recent studies demonstrated that MYCN overexpression increased the likelihood of NB cells undergoing ferroptosis [11–13]. This phenomenon also reveals the metabolic deficiencies correlated with MYCN-amplified NB. These findings offer new avenues for targeted therapy, providing a basis for developing treatments and improving prognoses. Therefore, the relevance of FGs in MYCN-amplified NB was investigated for identifying novel biomarkers.

In recent years, the scientific community has conducted research on ferroptosis-related gene-targeted therapies for MYCN-amplified neuroblastoma (NB), achieving significant progress. Jiang et al. discovered that p53 can sensitize cells to ferroptosis by suppressing the expression of SLC7A11, a key component of the cystine/glutamate antiporter, thereby inhibiting cystine uptake [19, 20]. Combined with evidence that p53 gene expression is regulated by MYCN [21], p53 can be considered a potential therapeutic target for inducing ferroptosis in MYCN-amplified NB. The Trump research team identified LRP8, a low-density lipoprotein receptor, as a critical inhibitor of ferroptosis in MYCN-amplified neuroblastoma, with its regulatory mechanism potentially linked to selenocysteine metabolism. Their findings demonstrated that inhibiting the SELENOP/LRP8 axis represents a novel and selective strategy to trigger ferroptosis, thereby limiting the growth of highly aggressive and treatment-resistant MYCN-amplified neuroblastoma cells, confirming LRP8 as a promising therapeutic target [22]. Additionally, studies have shown that cell division cycle 27 (CDC27), a core subunit of the anaphase-promoting complex, sensitizes cells to ferroptosis. The CDC27-ODC1 axis accelerates ferroptosis, potentially offering new therapeutic strategies for NB patients [23]. Furthermore, researchers have identified potential intervention targets through risk models. For instance, Zhao et al. proposed that the gene AURKA could serve as a prognostic biomarker for NB [24], while Liu et al. suggested that EIF2S1, by regulating the expression of GPX4 and SLC7A11, may act as a potential therapeutic target for inducing ferroptosis in NB cells [25]. These studies collectively provide new insights and hope for improving treatment outcomes and prognosis.

This study analyzed neuroblastoma samples from the TARGET dataset and identified four ferroptosis-related genes significantly associated with patient prognosis: TP53, OSBPL9, LIFR, and NRAS. A prognostic model was constructed based on these four genes, and its accuracy in predicting overall survival (OS) was validated by ROC curve analysis. The reliability of the model was further confirmed by Cox regression analysis of risk scores and calibration curves of the nomogram. Therefore, we propose that the newly established prognostic model can be utilized to predict the prognosis of MYCN-amplified neuroblastoma patients. TP53, a gene implicated in nearly all critical cellular activities, is regarded as the “guardian of the genome.” It is typically expressed as a rapidly degraded protein and, under various stress stimuli, initiates cell cycle arrest and apoptosis through both transcriptional and non-transcriptional mechanisms [26]. In the context of ferroptosis, p53 primarily inhibits cystine uptake by downregulating SLC7A11 expression, thereby reducing glutathione (GSH) synthesis and inducing ferroptosis [20]. However, in certain cellular contexts and specific environments, p53 enhances GPX4-mediated resistance to ferroptosis by increasing GSH synthesis, thereby delaying or inhibiting ferroptosis [27, 28]. Although TP53 exhibits a dual role in regulating cancer cell sensitivity to ferroptosis, targeting the p53-ferroptosis pathway remains a superior therapeutic option compared to other targeted factors, given the potent tumor-suppressive functions of p53 across various cancers [29]. In neuroblastoma (NB), p53 is predominantly wild-type and lacks tumor-suppressive capacity [30]. However, studies on MYCN-amplified NB have identified TP53 as one of the targets of MYCN, with its promoter binding to MYCN and concurrently upregulating several p53 regulatory genes such as MDM2 and PUMA [31], resulting in poor clinical event-free survival and overall survival [32]. In the cellular validation experiments of key genes conducted in this study, TP53 expression levels were significantly lower in MYCN-amplified SK-N-BE [2] cells compared to non-MYCN-amplified SH-SY5Y cells, a finding that appears contradictory to the bioinformatics analysis results.The same results were also found in the study by Tan et al. [33]. Studies have confirmed that the MYCN gene can regulate the expression of MDM2, which, as a direct transcriptional target of p53, feedback inhibits p53’s transcriptional activity and reduces its expression. This may be one of the reasons for the decreased TP53 levels detected in MYCN-amplified neuroblastoma cells [34]. Secondly, bioinformatics analysis data may originate from specific time points or tissue regions, with the detection scope covering all transcript isoforms, whereas qPCR experiments are based on primers targeting specific isoforms, potentially leading to inconsistent conclusions. Additionally, the growth environment of in vitro cultured neuroblastoma cell lines differs from that in patients, and combined with the high heterogeneity of neuroblastoma itself [35], further validation with more clinical data and samples is still required OSBPL9 belongs to the oxysterol-binding protein-like (OSBPL) family, which comprises essential transport proteins on cell membranes that facilitate molecular or signal exchange between organelles, primarily involved in cellular lipid and cholesterol metabolism [36]. It may participate in ferroptosis by regulating and integrating sterol and phospholipid metabolism [37]. In this study, LIFR, a leukemia inhibitory factor receptor, interacts with LIF to trigger multiple signaling pathways, including those promoting tumor progression and metastasis as well as inhibiting tumor proliferation and invasion. However, research on targeted therapies for LIF/LIFR signaling remains insufficient [38]. Recent studies have identified LIFR as a tumor suppressor gene in hepatocellular carcinoma, where it inhibits the NF-κB signaling pathway by interacting with SHP1, thereby altering the expression of the target gene LCN2 and modulating sensitivity to ferroptosis, suggesting a potential avenue for targeted induction of ferroptosis [39]. NRAS, a proto-oncogene of the RAS family, was initially discovered in neuroblastoma [40] and drives ferroptosis through lipid metabolism regulation [41], emerging as a novel prognostic marker [42].

By applying the Cibersort algorithm, an in-depth analysis of ICI was performed. In the HR group, it revealed a notable decline in levels of memory B cells and resting memory T cells CD4. A recent study demonstrated that the majority of ICs, including CD4 and CD8 cells, exhibit low infiltration in HR groups, indicating immunosuppression [43]. The finding was in line with the conclusion of this study. Moreover, additional studies have validated that the anti-tumor function of T cells can be enhanced by inhibiting ferroptosis [44], a process with potential for immunotherapy.

In addition, limitations should also be noted in this study. Firstly, the prognosis model was constructed and verified using retrospective data from public databases, which required more datasets to verify its predictive effect. Additionally, the verification of gene expression levels in the experiments was not completely consistent and was not verified in more NB cells. Furthermore, due to the lack of clinical samples, the specific role of each gene in MYCN-amplified NB and ferroptosis-related studies remains insufficient, which is a valuable area for further exploration.

## Conclusion

In conclusion, a prognosis model comprising FGs was successfully developed in this study, and database internal validation has proved its good prediction performance. Furthermore, cell experiments have also validated the differential expression of genes, which is potentially relevant for further screening of intervention genes targeting ferroptosis. These findings offer a promising avenue for further studies on the intrinsic mechanisms of MYCN-amplified NB.

## Electronic supplementary material

Below is the link to the electronic supplementary material.

Supplementary Fig. S1 Multivariate COX Regression Analysis and Nomogram. (**A**) Multivariate COX regression analysis showing that risk scores based on characteristic genes are independent predictors of OS. (**B**) Establishment of a nomogram for prediction of patients’ 1-, 3-, and 5-year OS according to clinical characteristics and risk scores. Based on the variable values of each patient, draw an upward vertical line to calculate the respective values, summing them up as the “total score,” and then draw a downward vertical line to calculate the survival probabilities and median survival times at 1 year, 3 years, and 5 years

Supplementary Fig. S2 Enrichment Analysis of MYCN-related DEGs (**A**) Bubble diagram for GO enrichment analysis; (**B**) Bubble diagram for KEGG enrichment analysis. The circle size represents the number of genes enriched

Supplementary Fig. S3 Proportion and Difference in IC Composition between HR and LR Groups in TARGET Database (**A**) Bar stacking plot showing the proportion of 22 types of immunoinfiltrating cells in samples; (**B**) Boxplot showing the abundance of 22 types of ICs in the HR and LR groups

Supplementary Fig. S4 Verification of Key Gene RNA Expression Levels (**A**-**D**) Differential expression of NRAS (**A**), OSBPL9 (**B**), TP53 (**C**), and LIFR (**D**) genes in the database samples between MYCN-amplified and MYCN non-amplified groups (**E**-**H**) Differential expression of NRAS (**E**), OSBPL9 (**F**), TP53 (**G**), and LIFR (**H**) genes in MYCN-amplified and MYCN non-amplified NB cells in the QPCR experiment

Supplementary Table S1– Gene relative expression level

## Data Availability

The datasets generated during and/or analysed during the current study are available from the corresponding author on reasonable request.
