# Supplementary figures and images for: Construction of a ferroptosis-based prediction model for the prognosis of MYCN-amplified neuroblastoma and screening and verification of target sites
Source: Hereditas. 2025 Mar 19;162:41. doi: 10.1186/s41065-025-00413-8 (PMC11921587; doi:10.1186/s41065-025-00413-8)

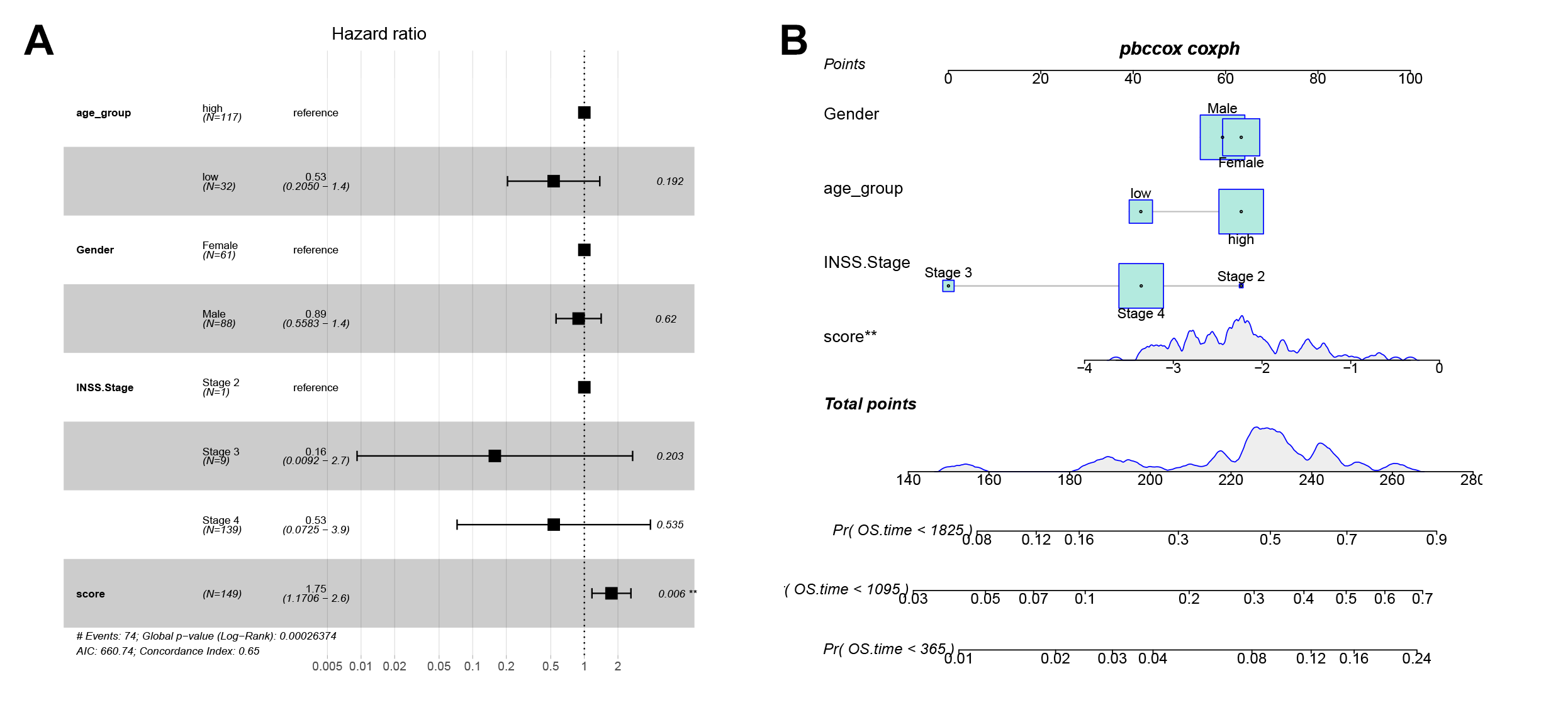

Supplement: Supplementary file 1 — Supplementary Fig. S1 Multivariate COX Regression Analysis and Nomogram. (A) Multivariate COX regression analysis showing that risk scores based on characteristic genes are independent predictors of OS. (B) Establishment of a nomogram for prediction of patients’ 1-, 3-, and 5-year OS according to clinical characteristics and risk scores. Based on the variable values of each patient, draw an upward vertical line to calculate the respective values, summing them up as the “total score,” and then draw a downward vertical line to calculate the survival probabilities and median survival times at 1 year, 3 years, and 5 years [file 41065_2025_413_MOESM1_ESM.tif]

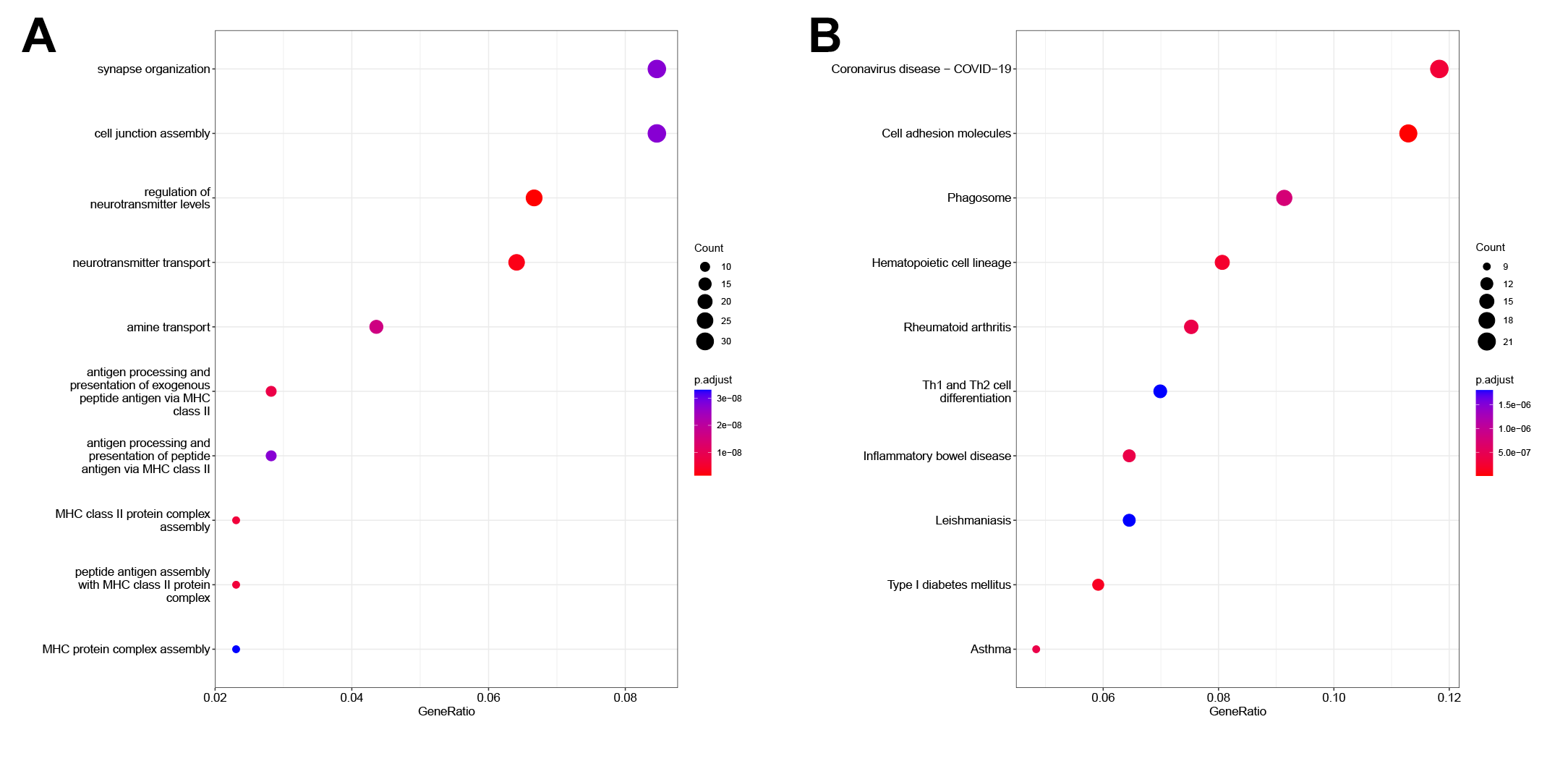

Supplement: Supplementary file 2 — Supplementary Fig. S2 Enrichment Analysis of MYCN-related DEGs (A) Bubble diagram for GO enrichment analysis; (B) Bubble diagram for KEGG enrichment analysis. The circle size represents the number of genes enriched [file 41065_2025_413_MOESM2_ESM.tif]

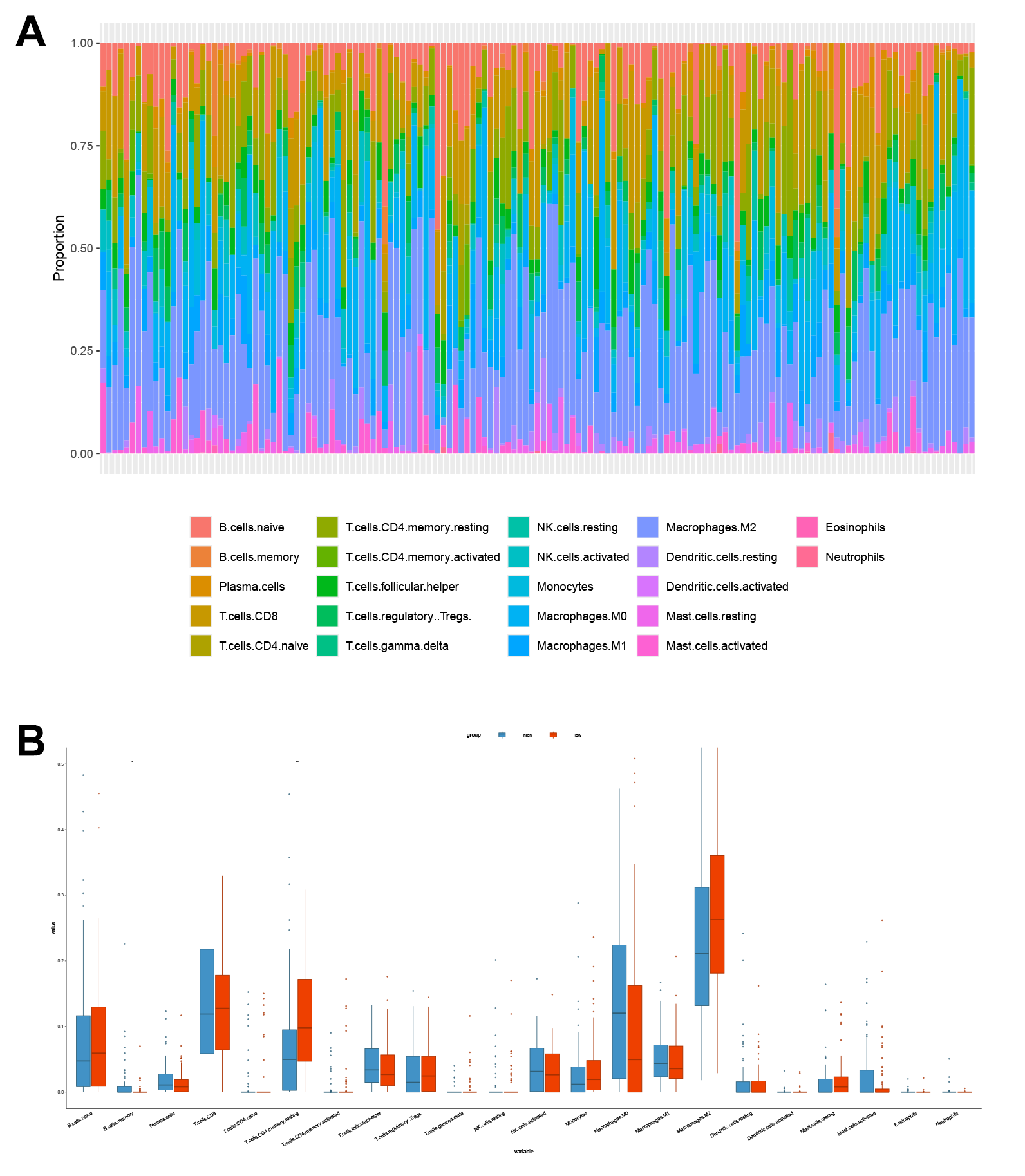

Supplement: Supplementary file 3 — Supplementary Fig. S3 Proportion and Difference in IC Composition between HR and LR Groups in TARGET Database (A) Bar stacking plot showing the proportion of 22 types of immunoinfiltrating cells in samples; (B) Boxplot showing the abundance of 22 types of ICs in the HR and LR groups [file 41065_2025_413_MOESM3_ESM.tif]

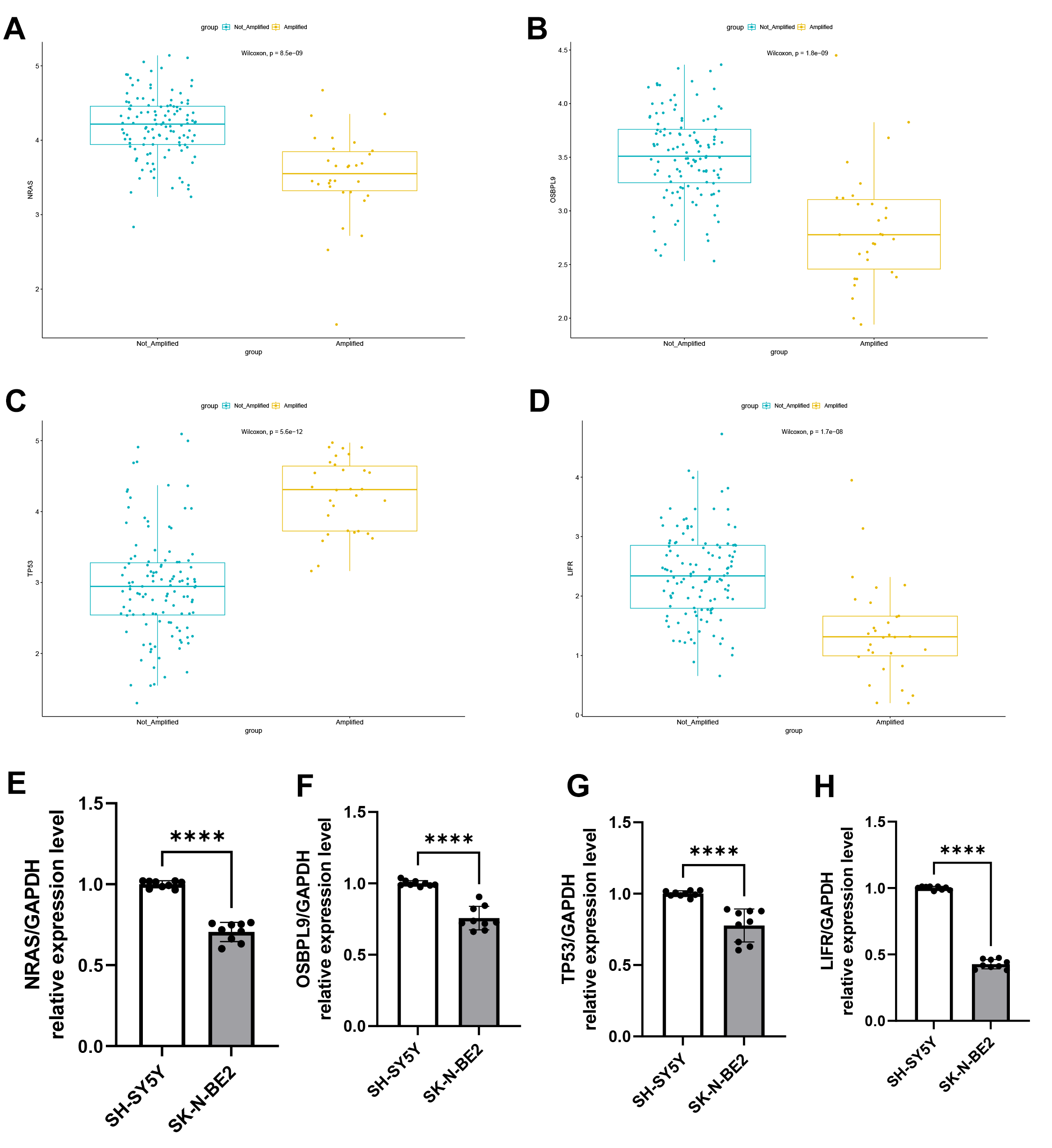

Supplement: Supplementary file 4 — Supplementary Fig. S4 Verification of Key Gene RNA Expression Levels (A-D) Differential expression of NRAS (A), OSBPL9 (B), TP53 (C), and LIFR (D) genes in the database samples between MYCN-amplified and MYCN non-amplified groups (E-H) Differential expression of NRAS (E), OSBPL9 (F), TP53 (G), and LIFR (H) genes in MYCN-amplified and MYCN non-amplified NB cells in the QPCR experiment [file 41065_2025_413_MOESM4_ESM.tif]
