# Supplementary material for: Construction of a ferroptosis-based prediction model for the prognosis of MYCN-amplified neuroblastoma and screening and verification of target sites
Source: Hereditas. 2025 Mar 19;162:41. doi: 10.1186/s41065-025-00413-8 (PMC11921587; doi:10.1186/s41065-025-00413-8)
Supplement: Supplementary file 5 — Supplementary Table S1– Gene relative expression level [file 41065_2025_413_MOESM5_ESM.docx]

Table S1 NRAS gene relative expression level

| SH-SY5Y | SK-N-BE2 |
| --- | --- |
| 0.97041 | 0.631418 |
| 1.018656 | 0.601513 |
| 1.011619 | 0.66281 |
| 0.965936 | 0.757858 |
| 1.021012 | 0.76313 |
| 1.013959 | 0.752623 |
| 0.986233 | 0.708742 |
| 0.993092 | 0.718636 |
| 1.021012 | 0.749154 |

Table S1 OSBPL9 gene relative expression level

| SH-SY5Y | SK-N-BE2 |
| --- | --- |
| 0.990801 | 0.670511 |
| 0.990801 | 0.66128 |
| 1.018656 | 0.718636 |
| 1.03766 | 0.721965 |
| 0.974905 | 0.726986 |
| 0.988514 | 0.737135 |
| 1 | 0.844791 |
| 0.986233 | 0.82169 |
| 1.013959 | 0.905425 |

Table S1 TP53 gene relative expression level

| SH-SY5Y | SK-N-BE2 |
| --- | --- |
| 1.023374 | 0.80478 |
| 0.988514 | 0.782773 |
| 0.988514 | 0.862542 |
| 1.023374 | 0.604299 |
| 1.016305 | 0.66128 |
| 0.961483 | 0.629961 |
| 1.006956 | 0.878633 |
| 1 | 0.890899 |
| 0.993092 | 0.878633 |

Table S1 LIFR gene relative expression level

| SH-SY5Y | SK-N-BE2 |
| --- | --- |
| 0.983957 | 0.411796 |
| 1.011619 | 0.406126 |
| 1.004632 | 0.417544 |
| 0.988514 | 0.381565 |
| 1.009285 | 0.441351 |
| 1.002313 | 0.384219 |
| 0.97716 | 0.461158 |
| 1.011619 | 0.467596 |
| 1.011619 | 0.474123 |
